# Efficacy and safety of Dingkun pill for female infertility patients with low prognosis undergoing in vitro fertilization-embryo transfer: study protocol for a multicenter, double-blind, randomized, placebo-controlled trial

**DOI:** 10.1186/s13063-020-04502-z

**Published:** 2020-06-19

**Authors:** Jingyan Song, Ting Ma, Yanlin Liang, Xianling Cao, Zhengao Sun

**Affiliations:** 1 The First Clinical College, Shandong University of Traditional Chinese Medicine, Jinan, 250014 China; 2 Reproductive and Genetic Center of Integrated Traditional and Western Medicine, The Affiliated Hospital of Shandong University of Traditional Chinese Medicine, Jinan City, 250011 Shandong Province China; 3 Science and Technology Department, Shandong University of Traditional Chinese Medicine, Jinan, 250300 China; 4 Medical Marketing Department, Shanxi Guangyuyuan Medicine Co., Ltd., Xian, 710003 China

**Keywords:** Traditional Chinese medicine, Dingkun pill, In vitro fertilization, Diminished ovarian reserves, Poor ovarian response

## Abstract

**Background:**

Approximately 15% of couples in the reproductive age are affected by infertility. Women with diminished ovarian reserves (DOR) or with a poor ovarian response (POR) are required to undergo in vitro fertilization and embryo transfer (IVF-ET) to achieve pregnancy. However, studies indicate that poor response to gonadotropin stimulation has been reported in women undergoing IVF-ET. Results from two recent clinical studies in China suggest that traditional Chinese medicine (TCM) formula Dingkun pill (DKP) showed a curative effect by improving the clinical pregnancy rate in women with DOR and POR. However, the heterogeneity of the studies does not allow one to draw a definitive conclusion on the therapeutic effect of DKP. Therefore, the purpose of this study was to investigate the effect of DKP on improving the clinical outcome of pregnancy of IVF-ET in women with low prognosis.

**Methods:**

A multicenter, double-blinded, randomized placebo-controlled trial was conducted. A total of 460 infertile patients undergoing IVF or intracytoplasmic sperm injection (ICSI) were recruited from 12 public hospitals in China. Participants were randomly divided into the experimental group (DKP formula) or the placebo group (control) at a ratio of 1:1. All patients were treated with GnRH antagonist protocol and ovarian stimulation performed for 5 weeks (from the 5th day of the previous menstrual cycle to the day of oocyte retrieval). The patients were followed up for 6 months to record their conception outcome. The primary outcome is to compare the pregnancy outcome to those under placebo treatment. Secondary outcomes included the total count of the retrieved oocyte, embryo quality, endometrial thickness on ET day, implantation rate, and early miscarriage rate.

**Discussion:**

Currently, no multicenter, double-blind, randomized, placebo-controlled trials have been performed on the use of the DKP formula to improve on the clinical outcome of the conception of IVF-ET in women with low prognosis. DKP might provide a good clinical solution for females with low prognosis and undergoing IVF. There is no contemporary Western medicine to improve on the clinical outcome of conception in IVF-ET in women with low prognosis. Therefore, it is important to undertake a well-designed randomized trial to determine the effect of DKP in improving the clinical outcome of the conception of IVF-ET in women with low prognosis.

**Trial registration:**

Chinese Clinical Trial Registry (ChiCTR). Trial registration number: ChiCTR1900026614. Registered on 16 October 2019.

## Background and rationale

Infertility affects approximately 15% of couples of reproductive ages and female infertility is a global reproductive health problem [1]. Currently, postponing childbearing due to socio-economic reasons has shown an increasing trend worldwide. The consequences of this are an increased number of women having a poor ovarian response (POR) prior to IVF due to their old age. Women in their mid to late thirties are reported to more likely have diminished ovarian reserves (DOR) due to irreversible intrinsic aging of the ovaries, and this highlights the need to focus on this group of women undergoing IVF-ET [2, 3]. Even though IVF-ET has become an effective and widely available treatment for infertile couples, women with DOR or POR to exogenous gonadotropin stimulation present a challenge to reproductive experts [4].

Low prognosis is one of the most intractable problems of IVF-ET treatment, occurring in approximately 47% of women with DOR, and 55% of these women aged ≥ 35 years [5–7]. Patients with low prognosis require not only an increased dose of gonadotropin (Gn) but also increased time for ovarian stimulation during an IVF cycle. However, there are a number of associated disadvantages such as fewer oocyte yield, poorer embryo quality, and pregnancy outcome [8]. These factors cause emotional, physical, and financial burden and distress for the couple, especially when multiple treatment cycles are required [9]. Regardless of the various pre-treatment strategies available including coenzyme Q10 [10] and dehydroepiandrosterone (DHEA) [11], there is a lack of sufficient evidence on the ability of these therapeutic agents to reverse low prognosis especially in women of advanced age and with DOR [12].

DKP is one of the traditional Chinese medicines first used during the reign of Emperor Qianlong of the Qing dynasty (A.D. 1636–1912) as a “holy medicine in the emperor’s harem”, and it was used exclusively by the imperial court. DKP components include ginseng, deer antler, safflower, *Caulis spatholobi*, *Radix rehmannia preparata*, *Angelica*, *Scutellaria*, *Rhizoma cyperi*, *Leonurus japonicus houtt*, ligustrazine, *Rhizoma corydalis*, and other 30 traditional precious Chinese herbs. It is commonly used in the treatment of several medical conditions such as irregular menstruation, dysmenorrhea, osteopyrexia and fever, leukorrhea with reddish discharge, and metrostaxis. DKP is used to tonify the liver and kidney, invigorating qi and nourishing blood, regulating menstruation, relaxation to combat depression, promoting blood circulation, and relieving pain. In Chinese medicine practice, TCM pathogenesis in elderly women with low prognosis is mainly manifested by spleen and kidney deficiency, blood deficiency, and liver depression, which is consistent with the TCM syndrome type of DKP. DKP is reported in previous studies to have a curative effect on increasing the clinical factors affecting pregnancy rate in women with DOR and POR [13, 14]. However, the heterogeneity of these studies does not provide definitive conclusions about the therapeutic effects of DKP, mainly due to the use of small sample size, unknown randomization methods, inconsistent inclusion criteria, and lack of a placebo group. Therefore, it is important to carefully design a randomized controlled trial to confirm the efficacy of DKP pills in improving the clinical outcome of the conception of IVF-ET in women with low prognosis.

## Methods

### Objective

The objective of our present study will be to investigate the effect of DKP pills on improving the clinical outcome of IVF-ET in women with low prognosis.

### Trial design and setting

**Fig. 1:**
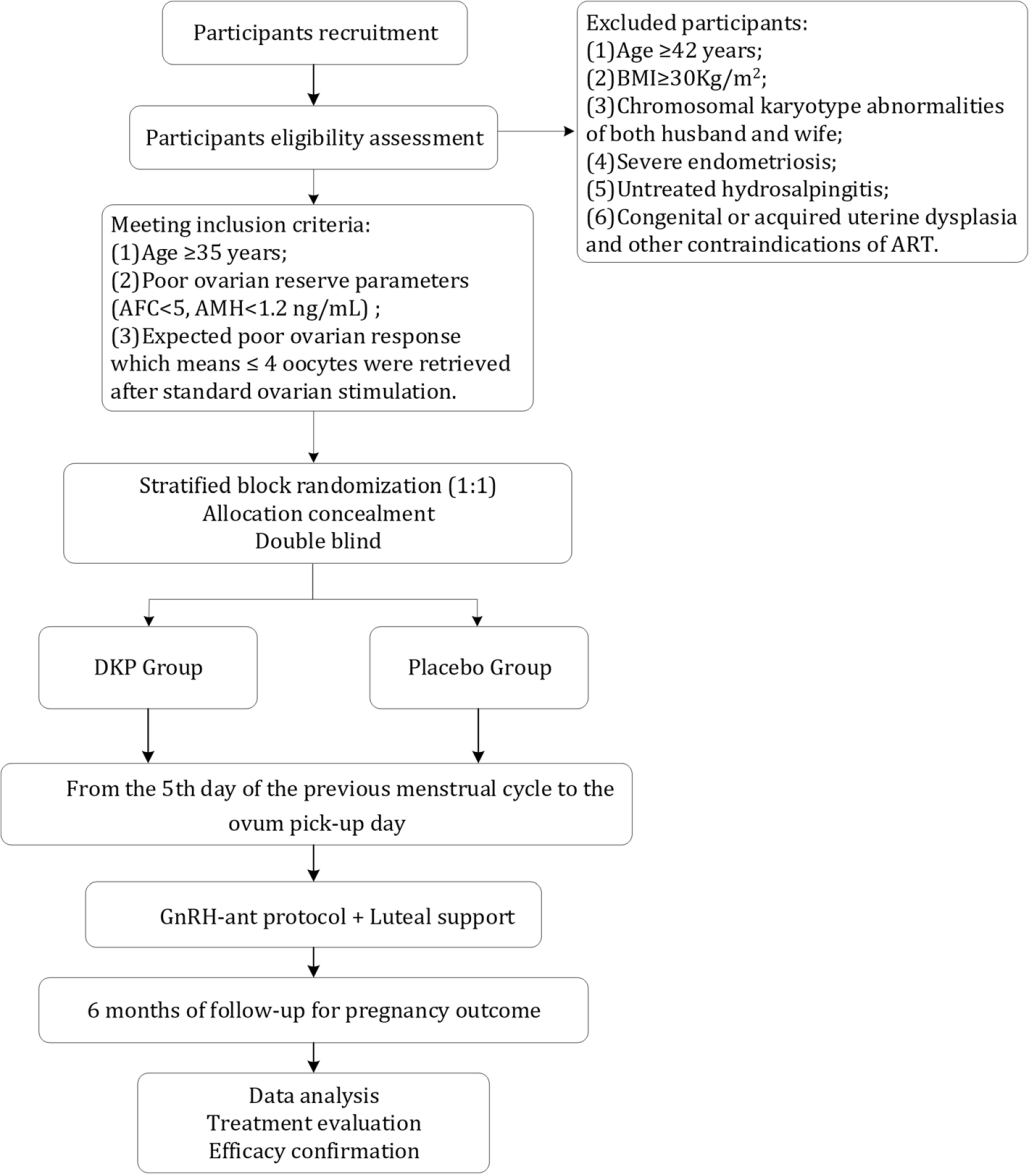
The study flowchart

This is a prospective, multi-center, randomized, double-blinded, placebo-controlled, superiority study. Eligible participants will be randomly assigned to the experimental group (DKP) or the control group with a 1:1 ratio. The participants will be recruited from 12 hospitals across mainland China. The study flow chart and schedule are shown in Fig. 1 and Table 1 (SPIRIT Figure), respectively.

**Table 1:** Schedule of the study process (SPIRIT Figure)

	Study period				
	Enrollment	Allocation	Post allocation		
Timepoint*	1st visit	0 week	1–6 weeks	7–12 weeks	4–6 months
**Enrollment:**					
Eligibility screen	**√**				
Informed consent	**√**				
Allocation by TCM team		**√**			
**Interventions:**					
DKP treatment			**√**		
Placebo treatment			**√**		
**Assessments:**					
Basic ovarian reserve test (AFC, bFSH, AMH, etc.)			**√**		
Laboratory index measurement (oocyte, zygote, embryo, etc.)				**√**	
Pelvic ultrasound evaluation (endometrium thickness and classification)				**√**	
IVF pregnancy outcome					**√**
Liver and renal function assessment				**√**	**√**

The main study site and coordinator are the Reproductive and Genetic Center of Integrated Traditional and Western Medicine and the Affiliated Hospital of Shandong University of TCM, respectively. The study is performed in collaboration with the Shandong University of TCM. The project team leader and main project coordinator have regularly communication and study visits have been performed at all study sites. In addition, meetings with all study sites are held annually or more frequently.

### Eligibility criteria

#### Inclusion criteria

Participants ≥ 35 years with poor pre-stimulation ovarian reserve parameters (AFC < 5, AMH < 1.2 ng/mL) and with an expected poor ovarian response (fewer than four oocytes) after standard ovarian stimulation will be recruited in this study.

#### Exclusion criteria

Patients will be excluded if their age ≥ 45 years, BMI ≥ 30 kg/m^2^, karyotyping abnormalities in both partners, endometriosis, hydrosalpinx, congenital or acquired dysplasia of the uterus, and other contraindications requiring assisted reproductive technology.

### Interventions

#### TCM DKP formula preparations

In traditional Chinese medicine, the decoction is prepared by boiling in water for hours. However, DKP (Lot No. Z20059003, Shanxi Guangyuyuan Medicine Co., Ltd., China) preparation will adopt the water-honeyed pill protocol according to the Chinese Pharmacopoeia (ChP) 2015 Edition standard [15] and ChP 2005 Edition. The “DKP water-honeyed pill” standard [16] is approved by the China Food and Drug Administration (CFDA). Each TCM bottle is filled with 7 g DKP.

The genuine medicinal materials are used in all kinds of traditional Chinese herbs, and the specific purchasing locations are stipulated as township-level sales points where genuine medicinal materials are located, which are purchased in the same batch. The medicinal materials are processed in strict accordance with the requirements, and standard operation procedures are formulated. The test results of drug quality were consistent with the Chinese Medicine Standards of the State Food and Drug Administration.

#### Placebo preparation

The placebo will be provided by Shanxi Guangyuyuan Medicine Co. Ltd. (China). The DKP placebo will be a mixture of 55% starch and 45% caramel mixed, dried, crushed, and lumped together. The daily doses of the placebo are packed in individual TCM bottles for easy consumption under the ChP 2015 Edition standard the Good Manufacture Practice of Medical Products (GMP) standard. Patients in the placebo group will consume the same amount of placebo as the treatment group.

The placebo and the herbal medicines used to make DKP are identical in appearance, color, smell, taste, packaging, usage, and dosage [17]. During the production of placebo, the selection of condiments, colorants, and other excipients should be carefully carried out, and strictly in accordance with the Chinese Medicine Standards of the State Food and Drug Administration.

#### Study process

Prior to the control of ovarian hyper-stimulation (COH), all participants will receive DKP 7 g twice daily on the 5th day of their menstrual cycle until the oocyte retrieval day.

##### COH

GnRH antagonist (cetrorelix; Merck Serono, Darmstadt, Germany) is administered subcutaneously at a daily dose of 0.25 mg when there is at least one follicle measuring ≥ 12 mm in mean diameter on the trigger day, with 150–450 IU/day of recombinant FSH (Puregon, MSD, Courbevoie, France; Gonal-F, Merck-Serono, Lyon, France) and urinary FSH (hMG, Menotrophin for Injection, Livzon Pharmaceutical Group Inc., Guangdong, China). Gonadotropin doses will be determined based on individual patient’s characteristics. Final oocyte maturation will be triggered when more than two ovarian dominant follicles measuring ≥ 18 mm are visible by ultrasound. Final oocyte maturation will be achieved using either a single 0.2 mg injection of GnRH agonist (Triptoreline, Decapeptyl, Ipsen, France) or 250 μg of recombinant hCG (rhCG, Ovitrelle, Serono, France). Oocyte retrieval will be performed after 35–36 h by transvaginal ultrasound-guided aspiration.

##### Oocyte retrieval and embryo culture

BD Falcon IVF medium (Becton, Dickinson and Company, Franklin Lakes, NJ, USA) will be used to collect the Oocytes and perform embryo culture. Incubation conditions will be set at 6% CO_2_, 5% O_2_, and 37.0 °C (C200 CO_2_ Incubator, Labotect Labor-Technik-Göttingen GmbH, Göttingen, Germany). Culture oocytes will be inseminated for IVF or decumulated for ICSI.

Transfer of two high-quality embryos will be performed on day 3, and the surplus frozen on day 3 or day 5 based on the routine at different sites. All good quality embryos will be cryopreserved via vitrification (CBS-ViT-HS, CryoBioSystem®, L’Aigle, France). Dimethylsulfoxide and ethylene glycol will be used as cryoprotectants (Irvine Scientific Freeze Kit®, Irvine Scientific, Newtown Mount Kennedy, Ireland, and Vitrification Kit 101, Cryotech®, Tokyo, Japan).

##### Endometrial preparation and embryo transfer

Artificial endometrial preparation will consist of sequential administration of E_2_ valerate and injectable progesterone. A total of 2 mg E_2_ valerate will be administered twice daily for 14–16 days, and the dose later adjusted based on the endometrial thickness measured by vaginal ultrasonography. For endometrial thickness ≥ 7 mm, injectable progesterone supplementation will be initiated while for endometrial thickness < 7 mm, the patients will continue taking oral E_2_ until the endometrium attains the required threshold. About 20 mg of progesterone will be administered and FET will be performed after 3 days. Ultrasound-guided soft catheter embryo transfers will be performed.

#### Adherence

Each study center will have a local coordinator responsible for a case report form (CRF) to ensure adequate recording of individual participant’s data. To improve data validity, several methods will be used to assess drug compliance, including TCM bottle count and weekly telephone follow-up. Unused medicines will be counted and recorded.

#### Concomitant care

All participants will not be allowed to take other Chinese herbal supplements or nutritional supplements to enhance ovarian reactivity for 3 months prior to their enrollment into the study, since they may affect the effectiveness of the DKP formula.

### Outcome measurements

#### Primary outcomes

The primary outcome is the ongoing clinical pregnancy rate, defined as the presence of intrauterine gestation sac with fetal heart rate at 7 weeks of gestation.

#### Secondary outcomes

Secondary outcomes include total retrieved oocyte count, embryo quality, endometrial thickness on ET day, implantation rate, early miscarriage rate, and safety assessment on the TCM formula. Early miscarriage is defined as the spontaneous loss of pregnancy within the first 13 weeks of gestation. Implantation rate is defined as the number of gestational sacs per the number of embryos transferred.

#### Retention

Upon recruitment, a research assistant will conduct weekly follow-ups to remind the participant of upcoming doctor/data collection appointments and the recording of any adverse drug effects.

### Statistical analysis

#### Sample size calculation

PASS software version 11.0 (NCSS, LLC. Kaysville, Utah, USA) will be used to calculate sample sizes for both groups. Two recent studies conducted in China report that the clinical pregnancy rates of POR patients receiving DKP treatment and undergoing conventional IVF or ICSI based on a micro-stimulation protocol are 39.62 and 20.93%, respectively [13, 14]. In the present study, the clinical pregnancy rate in the DKP group is hypothesized to be 0.4 based on the null hypothesis and 0.25 on the alternative hypothesis. The clinical pregnancy rate in the placebo group is hypothesized to be 0.25. The test statistic used is the two-sided *Z* test with pooled variance. The significance level of the test targeted is 0.05. The ratio between groups will be 1:1. The minimum sample size for each group will be 203; hence, a total of 406 participants will achieve 90% power to detect a difference between the group proportions of 0.15. Assuming that the dropout rate of study participants is 10%, we expect to recruit 460 participants, with 230 participants in each group.

#### Randomization and allocation concealment

Patients will be randomly divided into two groups (1:1 ratio) using computer-generated random numbers managed by the Integrative Medicine team. CARDS identifying the DKP group and the placebo group will be placed in sealed, opaque envelopes and distributed by designated professional managers. Both medications (DKP formula and placebo) will be prepared in a manner that they will appear similar in appearance, flavor, and smell. Blinding of the clinicians, patients, and outcome and data analysts will be done. To ensure high accuracy, the trial will follow the updated guidelines from the Consolidated Standards of Reporting Trials (CONSORT) 2010 Statement [18] and the SPIRIT-TCM Extension 2018 for reporting of parallel-group randomized clinical trials [19]. Un-blinding will only occur in exceptional circumstances when matters cannot be resolved with ongoing randomization, and this will be performed by an authorized investigator. All un-blinding events will be reported and their reasons are given on the corresponding CRF.

#### Data management

Upon meeting the inclusion criteria, patients will be informed of the project objectives by the reproductive medicine practitioner and presented with informed consent. The patient’s demographic information will be recorded on the CRF. Any errors will be crossed, corrected, and signed by the corresponding investigator. CRF data will be entered and coded to a corresponding e-CRF using the double-entry method. Hard copy CRFs will be kept in locked filing cabinets accessible to only authorized investigators. The e-CRFs will be stored in an encrypted data format in the server using the Advanced Encryption Standard, with access restricted to only authorized investigators. Data confidentiality will be guaranteed by minimizing the number of personnel handling the data.

#### Quality assurance

A Data Monitoring Committee (DMC) has been established. The DMC consists of an independent director from the Clinical Research Management Office of Shandong University of TCM, an independent director from the reproductive center of the affiliated hospital of Shandong University of TCM, and an independent director from the school of Information Management of Shandong University of TCM. DMC will be responsible for data monitoring, interim analysis, assessment of post-intervention adverse events and handling cases of liver and kidney dysfunction, review of core trial procedures and documentation, discussion of any revisions to the main study protocol, and making the final decision to terminate the trial.

#### Data dissemination

Study results will be disseminated in Open Access, peer-reviewed journals and shared through oral and poster presentations at international conferences. All resources (research summaries, training tools, manuals, etc.) will be uploaded to an online knowledge management platform.

#### Data analysis

Statistical analyses will be performed using SPSS software version 26.0 (IBM Corp, Armonk, NY). Kolmogorov-Smirnov test will be used to test the normality of the data. Normally distributed continuous variables are reported as mean ± SD, while non-normally distributed continuous variables are reported as median and IQR. Categorical variables are reported as absolute and relative percentage frequencies. For comparison of the baseline characteristics and outcomes, independent Student’s *t* test or the Mann-Whitney *U* test are used to evaluate the mean or median differences between the placebo and DKP groups for normally distributed continuous variables, and the Pearson *χ*^2^ test for categorical variables.

Multiple imputation will be used to process missing values in the data of this study. The intention-to-treat analysis is used to examine the differences in the clinical pregnancy rate for the two treatment arms in the primary analysis using the Pearson *χ*^2^ test. Similarly, the multivariate regression model is used to adjust the results based on potential confounding factors. All the tests are two-tailed, and the significance level is set as *p* < 0.05.

### Protocol amendments

Any modifications to the protocol which may affect the study conduct, potential benefits to the participants or participant’s safety including changes in study objectives, study design, patient population, sample sizes, or study procedures will require a formal amendment to the protocol. Such an amendment will be agreed upon by the DMC and presented to the Ethics Advisor and the relevant local ethical review bodies.

## Discussion

Low prognosis for pregnancy presents one of the most intractable problems in women undergoing IVF-ET treatment. This results in the need for more gonadotropin dosage and duration which is also associated with disadvantages such as fewer oocyte yield, poorer embryo quality, and unsatisfactory pregnancy outcome. In particular, women of advanced age (≥ 35 years) with poor ovarian reserve (AFC < 5, AMH < 1.2 ng/mL) are more likely to experience poor IVF treatment outcome. Furthermore, regardless of the available treatment strategies, no effective therapeutic agent is available to reverse low prognosis, especially in women of advanced age. Therefore, it is imperative to explore a potential therapeutic protocol for this population. DKP is a TCM formula that is efficacious in improving pregnancy outcomes in patients with a low prognosis; however, no RCT has been established to support this finding.

This RCT will be conducted by a group of experienced experts from different fields, including reproductive medicine, traditional Chinese medicine, and endocrinology. Based on our experience in conducting RCTs, this project will demonstrate that DKP has a convincing effect on alleviating various physiological and psychological stress and improving clinical outcomes in patients with low prognosis.

## Trial status

The trial was registered at the Chinese Clinical Trial Registry (ChiCTR) which is assigned to be the representative registry of China to join WHO ICTRP in 2007. This trial is at version 1.2, 16 October 2019 (ChiTR). The actual study start date was 1 October 2019 and the anticipated study end date is 31 December 2021. The recruitment start date was 15 June 2020; the anticipated recruitment end date is 31 December 2020.

## Supplementary information

**Additional file 1.**

## Data Availability

The datasets generated and analyzed during the current study are available from the corresponding author upon request.

## Abbreviations

RCT: Randomized clinical trial
ChP: Chinese Pharmacopoeia
GMP: Good Manufacture Practice of Medical Products
CFDA: China Food and Drug Administration
DMC: Data Monitoring Committee
CRF: Case report form
IVF-ET: In vitro fertilization-embryo transfer
FSH: Follicle-stimulating hormone
E_2_: Estradiol
TCM: Traditional Chinese medicine
WHO: World Health Organization
DKP: Dingkun pill
AFC: Antral follicle count
AMH: Anti-Mullerian hormone
DOR: Diminished ovarian reserve
POR: Poor ovarian response
