# Supplementary material for: Efficacy and safety of Dingkun pill for female infertility patients with low prognosis undergoing in vitro fertilization-embryo transfer: study protocol for a multicenter, double-blind, randomized, placebo-controlled trial
Source: Trials. 2020 Jun 19;21:550. doi: 10.1186/s13063-020-04502-z (PMC7304132; doi:10.1186/s13063-020-04502-z)
Supplement: Supplementary file 1 — Additional file 1. [file 13063_2020_4502_MOESM1_ESM.pdf]

山东中医药大学附属医院

中西医结合生殖与遗传中心（妇二科）

中药定坤丹对低预后女性体外受精-胚胎移植结局影响的随机对照研究

参与者知情同意书

我们已知 IVF 低预后患者的妊娠结局不是非常理想。我们仍未了解中国传统草药能否改善低预后患者的妊娠结局。这项研究的目的是探讨定坤丹对低预后女性 IVF 妊娠结局的影响和其中的机制。

根据 POSEIDON 标准，你隶属于低预后患者分类，如果你愿意参与这项研究，你需要：

I. 提供 5-10 毫升血液作生殖内分泌激素或生化检测。抽血的过程中会有很轻微的痛楚，及非常小的感染风险。

II. 花 5-10 分钟的时间填写问卷。

III. 服用其中一种药物 (a) 或 (b) 5-6 周（前一月经周期第 5 天至取卵当日）：

(a) 中药水蜜丸：每日用药均由单独药瓶分装，每瓶 7g。这其中包括红参、鹿茸、西红花、鸡血藤膏、白芍、熟地黄、当归、黄芩、香附、茺蔚子、川芎、延胡索等 30 味名贵中药材。服用方法：每次 7g，每日 2 次。这些中药并没有任何副作用。

或

(b) 安慰剂：和中药水蜜丸(a)的外形完全相同。

IV. 在服用完所有中药水蜜丸或安慰剂 3 个月内完成新鲜或冷冻胚胎移植，按照医嘱规定的节点定期复诊，行抽血或 B 超检查并记录临床结局。这些检查并没有任何风险。

V. 我们将会在您完成研究后的 6-12 个月致电给您，询问有关胎儿产科结局的情况。

您将授权山东中医药大学附属医院生殖医学伦理委员会查阅有关您涉及研究的资料，作为伦理审查。所有与此项研究有关的资料，均会保密处理。您是否参加此项研究，亦不会影响您应有的治疗。如您对此项研究有任何问题及查询，请致电山东中医药大学附属医院孙振高副教授（电话：0531-68617921）。若你对参与此研究的权利有疑问，可于办公时间上午 8 点至下午 5 点向山东中医药大学附属医院生殖医学伦理委员会查询（电话：0531-68617980）。

若您现在充分了解有关此项研究的资料而又决定参与，请您在同意书上签名。您的参与全是自愿性质。在签署同意书后，您仍有权随时放弃参与此项研究。您是否参与此项研究，亦不会影响您应有的治疗。

本人同意参与上述研究。本人明白：

✓ 参与此项研究纯属自愿；

✓ 本研究不涉及生物样本的获取和保存；

✓ 本人有权随时放弃此项研究；

✓ 所有资料会保密处理，并只作研究用途；

✓ 本人同意*/不同意*与参与研究的机构和监管部门共享临床资料（*请删除其中 1 项）；

✓ 若中途退出，本人同意*/不同意*提供临床资料，并将有关数据存储 10 年（*请删除其中 1 项）。

受试者签名： 联系电话： 日期： 年 月 日

研究者签名： 联系电话： 日期： 年 月 日
